# The Influence of Innovation Climate on Creative Role Identity: The Mediating Role of Flow

**DOI:** 10.3389/fpsyg.2022.866464

**Published:** 2022-05-24

**Authors:** Baijun Deng, Jijuan Cao, Jieqi Huang, Jun Wu

**Affiliations:** ^1^School of Innovation and Entrepreneurship, Guangzhou Panyu Polytechnic, Guangzhou, China; ^2^Programs’ Development Department-DBA Office, Montpellier Business School, Montpellier, France

**Keywords:** innovation climate, creative role identity, flow, situational strength theory, entrepreneurship education

## Abstract

Creative role identity is an important antecedent of innovative behaviors. Both the mechanism of how external factors and individual factors affect the formation of creative role identity and details of this process have yet to be discovered. Based on data collected from 226 students in 6 classes at a university in Guangdong during the development of innovative projects, the influence of innovation climate on the students’ creative role identity, especially the mediating effect of flow, was investigated. The results show that the innovation climate has a positive impact on creative role identity and that flow plays a partial mediating role in this relationship.

## Introduction

How to stimulate individual innovation behavior has become an important issue faced by researchers and practitioners. The turbulence brought about by the COVID-19 epidemic has made enterprises increasingly need colleges and universities to cultivate talents who can actively innovate and tolerate high uncertainty to ensure the competitive advantage in an uncertain environment. Many studies have shown that creative role identity is an important antecedent of the encouragement of individuals to produce innovative behaviors (Wang and Cheng, 2010). Farmer et al. (2003) applied role identity theory to the field of creativity research and proposed the concept of creative role identity to describe the extent to which employees define themselves as creative individuals. The concept was subsequently followed by researchers. The significance of creative role identity is that individuals consciously transform external expectations and role requirements into a part of their self-awareness through identity adjustment mechanisms, and try their best to keep their attitudes and behaviors consistent with their social roles (Wang and Cheng, 2010). To facilitate the innovative behaviors of individuals, organizations must understand how employee’s creative role identities are formed.

However, existing studies mainly focus on the impact of self-factors on the creative role and seldom focus on the antecedents caused by external factors. Since 1950, when Guilford called on researchers to focus on the importance of creativity in education and other social domains, research on creativity in academia has increased significantly (Runco, 2004) and has focused on people’s “inner self,” emphasizing the recognition of personal attributes of knowledge and creativity (Finke et al., 1992). Human-centered research disconnects individual creativity from its environment. However, the complexity of human behavior means that individual behavior cannot be studied in a vacuum, and the interactive effects of individual differences and situational characteristics must be considered simultaneously (Mischel and Shoda, 1999). In fact, creativity is a “situational” activity (Loi and Dillon, 2006). According to situational strength theory (Meyer et al., 2010), the situational strength of an individual’s organization (such as a leader’s behavioral style and organizational climate) provides important external cues for the suitability of an individual’s specific behavior/behavioral performance/behavioral intention. Psychological factors, such as individual traits, interests, values, cognition, and emotions, work together to significantly hinder (or promote) the transformation of specific behaviors, behavioral performance, and behavioral intentions.

Innovation climate, as an important environmental factor affecting innovation willingness and innovation behavior, has always been a concern of the academic community (Choi, 2004; Eisenbeiss et al., 2008). However, there is still no clear conclusion on how innovation climate affects creative role identity, and further theoretical demonstration and empirical testing are needed (Newman et al., 2020).

According to situational strength theory, situational strength affects behavior/intentions through individual psychological factors. Flow is a construct in positive psychology, proposed by Csikszentmihalyi (1988), which means that people may experience a unique sense of flow, and move into a state in which they feel excited, have a sense of enjoyment, and are bursting with amazing creativity. Therefore, in the process of considering the influence of innovation climate on creative self-identity, we choose flow as a mediating variable to try to open the “black box” of the above process.

This paper takes innovative education in the field of pedagogy as the research background. From the perspective of pedagogy, the “disease” that stifles creativity in traditional classrooms has afflicted students for a long time. In this paper, a view of constructing a new way of classroom organization at the school and teacher levels to find an “unconventional learning climate” (Tan, 2001) that stimulates students to learn and explore through active and wholehearted participation is presented (Tan, 2001; Kampylis, 2006). The aim is that students could not only express their own creativity, but also become systematically more creative, and that ultimately all learners could attain creative achievement.

In our research objects—innovation and entrepreneurship courses at a university in Guangdong, China—students develop a complete innovation project in a relatively short period of time. This project engages students in a miniature version of the innovation activities within organizations, which is very beneficial when studying individual innovation behavior in different organizational innovation climates (in this study, different classes). Based on the data collected by 226 students in 6 classes in a Guangdong university during the development of innovative projects, this study put the innovation climate and creative role identity theory and the flow theory in psychology into the situation of classroom teaching for discussion and then attempts to reveal the key path through which the innovation atmosphere affects individual creative role identification.

In general, from the perspective of situational strength theory, this study analyzes the psychological mechanism of the influence of innovation climate on individual creative role identity. This paper makes the following contributions to the theory. First, it studies the antecedents of creativity role identity, introduces situational strength theory, and provides a new perspective for research on creativity role identity. Second, it proposes and verifies the influence mechanism of the external environment on role identity of creativity and discovers the key influence of flow as a psychological mechanism in this process. Third, this study is an extension of the research on relationship between external atmosphere and individual role identity in the context of pedagogy and provides useful new insights into educational methods improvement. The above theoretical contributions will also have important practical implications for enriching the teaching methods of colleges and universities in China and then promoting the cultivation of innovative talent.

## Hypothesis Development

### Innovation Climate and Creative Role Identity

The concept of organizational climate originates from the study of the psychological climate in the field of psychology. It is generally used to describe the dynamic and complex relationship between environmental stimuli and human behavior (Litwin and Stringer, 1968). Amabile et al. (1996) defined the organizational innovation climate as the degree to which individuals perceive support for creativity and innovation in their organizational environment, including factors that encourage creativity, autonomy and freedom, resources, and pressure, as well as barriers to creativity. The encouragement of creativity (including organizational encouragement, supervisor encouragement, and team encouragement), the degree of autonomy, access to adequate resources, and the opportunity for challenging work have positive effects on employees’ creativity, whereas workload and organizational barriers have negative effects. Existing research has noted that innovation climate and individual creative behavior (Jaiswal and Dhar, 2015), innovation behavior (Bain et al., 2001; Antoni, 2005), and knowledge sharing behavior (Edú-Valsania et al., 2016) have a significant positive correlation. Zhou et al., (2021) considered innovation climate as a situational factor in the process of exploring the influence of individual creativity role identity on innovation behavior and believed that innovation climate played a positive moderating role. The above studies have contributed to research on the influence of innovation climate on innovation behavior, but broader study of its process mechanism and empirical tests is still needed.

Role identity theory is widely used in social psychology and sociology to explain the causes of behavior. Role identity theory emphasizes that the self is the object to be recognized, the role is the element that constitutes the self, and the self contains multiple roles. The formation of roles depends on the environment; the environment provides the basic conditions for the role to be shaped and affects role identification through three forces (Charng et al., 1988): (1) social network and social structure (Stryker, 1980), (2) social support (Turner, 1968), and (3) positive representations of individual roles by important people in the environment (Erez and Earley, 1993).

Creative role identity reflects employees’ self-concept in their perception of innovation and innovative behavior in their organization; thus, it is an important psychological factor that affects innovative behavior (Farmer et al., 2003). As a product of value judgment, the behavioral decision-making that drives individuals’ creative role identity has a positive impact on their intrinsic motivation to display innovative behaviors.

As Scott and Bruce (1994) indicated, organizational innovation support includes support for innovation from the organization, superiors, and colleagues. This support is also an important part of the organizational innovation climate (Amabile et al., 1996). In a strong innovative climate, individuals will perceive the support and encouragement of their superiors and colleagues for creative activities. When superiors encourage employees to carry out innovative behaviors, they express their expectations with positive supportive behaviors (Tierney and Farmer, 2004). This superior support affects employees’ intrinsic motivation and can influence their creativity by stimulating their intrinsic motivation (Amabile et al., 1996). Riley and Burke (1995) analyzed social relations and role identity and found that “colleagues” in work relationships have more direct communication opportunities with individuals at work, so colleagues’ expectations will shape employees’ creative role identity. Expectations are linked to supportive behaviors. For example, when superiors expect employees to be creative, they will express their expectations by exhibiting positive supportive behaviors (Tierney and Farmer, 2004). Therefore, it is reasonable to believe that the support of colleagues is very important for employees and that this has a direct impact on their creative role identification. Various types of social and emotional support within an organization help employees realize their creative roles, thereby stimulating their intrinsic motivation for creative work (Hirst et al., 2009; Wang and Cheng, 2010). This discussion suggests that an organization’s encouraging attitudes toward creativity and the specific supportive behaviors of superiors and coworkers both convey important situational support signals to employees that can help encourage them to form a creative role identity.

Based on the above analysis, we propose the first hypothesis:

*H1*: Innovation climate has a positive impact on creative role identity.

### Flow as a Mediator

Researchers have focused on the influence of innovation climate on individual innovation behavior for years. However, sociological theory generally holds that the factors that influence individual behavior are multifaceted. The impact of environmental factors on individual behavior may be complex. An example is the perspective of social cognition theory, which argues that motivating factors (including environmental factors) that influence human behavior may stimulate people to act by evoking core beliefs rooted in individuals (Glăveanu and Tanggaard, 2014). On this basis, Kang et al. (2016) found that team innovation climate promotes employees’ innovative behavior by enhancing employees’ enthusiasm for invention, and with the increase in proactive (risk-taking) climate, innovation climate and innovation enthusiasm (employee innovation) become stronger. Magni et al. (2018) found that team innovation climate promotes innovative practice by increasing individuals’ attitudes toward proactivity and risk-taking. Nevertheless, the process mechanism by which organizational innovation climate affects employees’ innovative behavior intentions/results is still unclear, and more comprehensive analysis and empirical tests are needed (Newman et al., 2020). For example, current research focuses too much on the role of individual factors (cognition, spirit) in the process of the innovation atmosphere affecting individual innovation behavior, ignoring the current state of the individual. In fact, from the perspective of pedagogy, focusing on whether the environmental atmosphere can promote individual actions by affecting the individual state is as important as mining the beliefs of the students themselves.

Therefore, we focus on an important concept in positive psychology—flow. Csikszentmihalyi (1988) coined the term “flow,” describing the concept as follows: “The state in which people are so involved in an activity that everything else seems irrelevant; the experience itself is so enjoyable, So much so that people will even pay a huge price for doing so.”

Following Csikszentmihalyi’s (1988) research on flow, Landhäußer and Keller (2012) concluded that people’s work performance is improved when they experience flow at work. The generation of flow can also improve an individual’s work engagement. Bakker (2005) proposed the concept of work-related flow and believed that the flow experience of individuals in the process of work mainly includes three elements: absorption, work enjoyment, and intrinsic work motivation. Absorption refers to a state of total concentration during which employees are completely immersed in their work. Enjoyment means that employees enjoy their work and feel happy. Intrinsic motivation refers to employees being motivated by the intrinsic aspects of their work tasks and wanting to continue their work.

Flow theory is widely used in the field of education. In research on the antecedents of flow, some scholars study human factors. Mesurado et al. (2016) pointed out that when students believe that they have autonomy in learning, they are more likely to have flow experience and participate more in learning activities; Hong et al. (2020) further discussed that learning motivation can stimulate the flow experience and then have a positive and direct impact on learning engagement (Özhan and Kocadere, 2020). Yoo and Kim (2018) found that students who like to discuss their work with others are more likely to present a report successfully, which makes students more likely to have a higher level of flow experience in the next teaching activity; Chen et al. (2017) emphasized that students with higher prosociality cooperate closely with others in a game, which enables team members to have a good flow experience; Yoo and Kim (2018) suggested that creating a moderate sense of tension during class can make students more focused. Other scholars have studied the influence of resource factors on flow. Vasiliou et al. (2014), Huang and Lin (2017), Pelet et al. (2017), Kim and Ko (2019), Fang and Huang (2021), etc., all considered that resource security, such as providing tablet computers and AR, is very important in their respective studies. It creates a sense of presence for the students and makes it easier for them to experience flow. Some scholars have also put forward innovative and interesting viewpoints on the influence of the curriculum setting, proving the important role of the practices of teachers and teacher support. For example, a dialog-style teaching method presented by two teachers makes the classroom more like a drama performance, which can make students focus better on class (Wang et al., 2021); the higher the level of simulation of real-world scenarios in practical courses, the timelier the feedback provided, and the more likely students are to experience flow (Yoo and Kim, 2018); a humorous climate can increase the frequency of flow experiences (Bartzik et al., 2021); the application of advanced teaching models and concepts in classroom teaching can make students more willing to devote themselves to learning and have more fun (Yang and Hsu, 2020). Some scholars have also studied the influence of task difficulty factors on flow in the classroom. Hung et al. (2015) believe that challenging game tasks are more likely to make people immerse themselves in activities, concentrate their energy, and experience enjoyment, thereby improving the flow experience. Demir and Seferoglu (2021) concluded in more detail that paired tasks reduce the task difficulty across the whole class so that students’ abilities and task difficulty are more closely matched, thereby making the flow experience better than single-person tasks. Therefore, we can conclude that the innovation climate in class, including teacher practice, teamwork setting, and teacher support, helps generate students’ flow experience.

The organizational innovation climate as perceived by individuals is their cognitive interpretation of the organizational innovation environment (Isaksen et al., 1999). Discussing only the direct influence of the innovation climate on creative role identity is not enough to clarify its influence mechanism. The innovation climate, which is an external factor, can only influence the formation of creative role identity by shaping an individual’s perception and psychological state. We believe that the key mediating factor explaining the formation process of creative role identity is flow, that is, an individual’s experience of immersing themselves in a climate of innovation and enjoying innovation.

Csikszentmihalyi (1988) pointed out three basic requirements for the generation of flow: first, clear goals and challenging tasks; second, timely feedback about results, timely and appropriate reinforcement, and support to encourage and increase the occurrence of creative behaviors; and third, a balance between the individual’s skills and the challenge level of the task. A task that is too challenging for the individual’s abilities will cause deep frustration and anxiety. A task that is not challenging enough will first be experienced as easy but will soon become boring (Hoffman and Novak, 1996). The challenging work dimension and resource support dimension of an innovative organizational climate satisfy the first and third points, while encouragement by the organization, superiors, and colleagues promotes the realization of the second point. Therefore, in organizations with a strong innovation climate, individuals are more likely to experience the generation of flow in innovation activities.

Flow experience leads to certain flow results (Csikszentmihalyi, 1988). In the field of education, the outcomes of flow are often individual and generally positive. Flow can improve students’ satisfaction and acceptance of the course (Mulik et al., 2020), making it easier to achieve the unity of knowledge and action; it can also enhance students’ determination to perform tasks, improve learning performance (Admiraal et al., 2011), make them enthusiastic about taking on more difficult tasks (Eisenberger et al., 2005), and ultimately promote their innovative behavior.

According to flow theory, when an individual is addicted to an activity and enjoys it, he or she will actively research it or explore it in great depth. This kind of focused, selfless, and playful spirit helps individuals break their routine, explore new things, and expand their divergent thinking and creativity, thereby contributing to the creation of creative role identities.

We thus pose our second hypothesis:

*H2*: Flow mediates the positive effect of organizational innovation climate on creative role identity.

## Methods and Data

### Sample and Data Collection

The importance of a school education in fostering students’ creativity has been proven (Cropley, 2001; Starko, 2017). Based on the research objectives of this paper, the survey adopts the method of convenience sampling. We focused on a sample of college students at a vocational university in China and selected a total of 226 students in 6 classes from a literature college and a science college. The survey was conducted toward the end of the innovation and entrepreneurship course to ensure that the students participating in the survey have just experienced the course fully and maintain a relatively deep experience of the course. We collected data through offline questionnaires. Before giving out the questionnaire, the teacher explained the purpose of this research and promised that the questionnaire would be used only for academic research. During the investigation process, the teacher did not guide the interviewed students on a subjective bias and left enough time for the students to complete the questionnaire. The teacher distributed 226 questionnaires, which were all valid. Among the 226 respondents, men accounted for 46% and women, 54%. Engineering majors accounted for 51.3%, with 36 students from the architectural engineering class and 80 students from the municipal administration class. The number of liberal arts majors accounted for 48.7%, with 82 students from the investment class and 28 students from the finance class. Table 1 summarizes the characteristics of the respondents.

**Table 1:** Characteristics of the respondents (*N* = 226).

		Frequency	Percentage
Gender	Male	104	46.0
	Female	122	54.0
Class	Architectural engineering	36	15.9
	Investment	82	36.3
	Finance	28	12.4
	Municipal administration	80	35.4

### Measurement

The measurement scales used in this study are mainly from the Western research literature. To ensure the validity of the measurements in the Chinese context, we adopted a translation and back translation procedure to revise the scales. The respondents used a 5-point Likert scale (1 = strongly disagree; 5 = strongly agree) to evaluate how well each item in the questionnaire described their situation.

We measured the respondents’ creative role identity (CRI) using the creative personal identity (CPI) scale alternative of Karwowski (2012). We retained all five items: (1) I think I am a creative person; (2) My creativity is important to who I am; (3) Being a creative person is important to me; (4) Creativity is an important part of myself; and (5) Ingenuity is a characteristic that is important to me.

We adopted Bakker’s (2008) flow scale, which consists of 13 items measuring three independent dimensions, namely, absorption (4 items), enjoyment (4 items), and intrinsic motivation (5 items).

To measure innovation climate, we mainly referred to Amabile’s KEYS (Assessing the Climate for Creativity) scale. As this study was conducted at a university, we adjusted the scale according to the specific circumstances, including incentive mechanisms, teacher practice, teamwork, teacher support, resource guarantee, organizational promotion, and self-directed work.

## Results

### Reliability and Validity

Table 2 demonstrates the descriptive statistics for the study, including the means, standard deviations, kurtosis, skewness, and PLS loadings of all 41 variables. All of the factor loadings were above the proposed level of 0.7 Hair et al. (2021), proving indicator reliability. Table 3 shows all of the constructs’ Cronbach’s alpha (CA) and composite reliability (CR) values, which surpassed the recommended threshold of 0.70, indicating sufficient reliability. The average variance extracted (AVE) values were higher than 0.5, confirming the convergent validity of the research data (Bagozzi and Yi, 1988). Discriminant validity was checked by a correlation test. Because the square root of each construct’s AVE was greater than its correlation coefficients with other constructs, discriminant validity was proven.

**Table 2:** Means, standard deviations, and PLS loadings.

Item	Mean	SD	Kurtosis	Skewness	PLS Loading
**Flow**					
*Absorption*					
ABS1	3.960	0.918	0.489	−0.827	0.820
ABS2	3.174	1.053	−0.637	0.039	0.804
ABS3	3.366	1.077	−0.603	−0.340	0.884
ABS4	3.567	0.989	−0.503	−0.299	0.932
*Work enjoyment*					
WE1	4.036	0.930	1.008	−1.044	0.910
WE2	4.049	0.960	0.846	−1.013	0.951
WE3	4.067	0.916	0.266	−0.835	0.960
WE4	4.022	0.938	0.982	−0.994	0.946
*Intrinsic work motivation*					
IWM1	3.964	0.944	1.177	−1.050	0.893
IWM2	3.598	1.035	−0.118	−0.545	0.782
IWM3	3.808	0.984	0.053	−0.654	0.899
IWM4	4.018	0.916	1.532	−1.088	0.801
IWM5	3.933	0.921	−0.218	−0.626	0.890
**Innovation climate**					
*Incentive mechanism*					
IM1	3.848	0.883	0.442	−0.675	0.895
IM2	3.929	0.873	0.655	−0.751	0.906
IM3	3.906	0.889	1.469	−0.966	0.912
IM4	3.946	0.864	0.765	−0.773	0.896
*Teacher practice*					
TP1	3.978	0.899	1.723	−1.108	0.913
TP2	4.076	0.870	1.727	−1.130	0.928
TP3	4.103	0.932	2.158	−1.338	0.944
*Teamwork*					
TEA1	3.929	0.913	0.945	−0.884	0.931
TEA2	4.000	0.881	1.363	−1.024	0.932
TEA3	4.036	0.891	0.372	−0.795	0.932
*Teacher support*					
TS1	4.018	0.930	2.112	−1.274	0.925
TS2	4.067	0.866	1.765	−1.127	0.894
TS3	4.062	0.899	1.001	−1.014	0.901
TS4	4.094	0.909	1.791	−1.229	0.893
*Resource guarantee*					
RG1	2.750	1.102	−0.518	0.267	0.979
RG2	2.763	1.078	−0.514	0.225	0.938
RG3	2.710	1.122	−0.493	0.286	0.882
*Organization promotion*					
OP1	3.826	0.941	1.199	−0.973	0.835
OP2	3.929	0.908	1.385	−1.009	0.940
OP3	4.040	0.965	1.471	−1.191	0.887
*Self-directed work*					
SDW1	3.594	1.000	0.092	−0.516	0.870
SDW2	3.759	0.894	1.135	−0.789	0.909
SDW3	3.612	0.943	0.087	−0.406	0.904
*Creative role identity*					
CRI1	3.554	0.953	−0.057	−0.341	0.785
CRI2	3.911	0.907	1.166	−0.979	0.884
CRI3	3.897	0.903	0.081	−0.638	0.876
CRI4	3.728	0.917	0.322	−0.551	0.903
CRI5	3.571	1.002	−0.478	−0.211	0.809

CRI, Creative Role Identity; IC, Innovation Climate; TS, Teacher Support; IWM, Intrinsic Work Motivation; ABS, Absorption; TEA, Teamwork; WE, Work Enjoyment; IM, Incentive mechanism; OP, Organization Promotion; SDW, Self-directed Work; RG, Resource Guarantee; TP, Teacher Practice.

**Table 3:** Reliability, validity, and corrections of the constructs.

	Cronbach’s alpha	CR	AVE	ABS	CRI	IM	IWM	OP	RG	SDW	TEA	TP	TS	WE
ABS	0.883	0.920	0.742	0.862										
CRI	0.906	0.930	0.727	0.606	0.853									
Flow	0.956	0.962	0.661	0.861	0.718									
IC	0.957	0.964	0.569	0.659	0.771									
IM	0.924	0.946	0.814	0.542	0.685	0.902								
IWM	0.906	0.931	0.730	0.728	0.717	0.661	0.854							
OP	0.866	0.918	0.789	0.529	0.675	0.760	0.725	0.888						
RG	0.932	0.953	0.872	0.106	0.163	0.064	0.080	0.069	0.934					
SDW	0.875	0.923	0.800	0.649	0.789	0.640	0.716	0.684	0.154	0.894				
TEA	0.924	0.952	0.868	0.568	0.663	0.707	0.661	0.714	0.048	0.686	0.932			
TP	0.920	0.949	0.862	0.620	0.643	0.805	0.715	0.757	0.023	0.638	0.788	0.929		
TS	0.925	0.947	0.816	0.616	0.678	0.790	0.738	0.797	0.039	0.698	0.820	0.880	0.904	
WE	0.957	0.969	0.887	0.703	0.643	0.652	0.853	0.685	0.050	0.646	0.693	0.746	0.757	0.942

*AVE, Average Variance Extracted; CR, Composite Reliability*.

To prove the internal consistency reliability, the values of CR should be higher than 0.7, and the values of average variance extracted (AVE) should be higher than 0.5 to confirm the convergent validity (Bagozzi and Yi, 1988). In this study, the values of CR are 0.964, 0.967, and 0.936 for flow, IC, and CRI, respectively, and the values of AVE are 0.677, 0.592, and 0.746 for flow, IC, and CRI, respectively. The reliability and validity of the measurement scales have been confirmed. Table 3 presents the CRs and AVEs.

### Testing of Hypotheses

**Figure 1:**
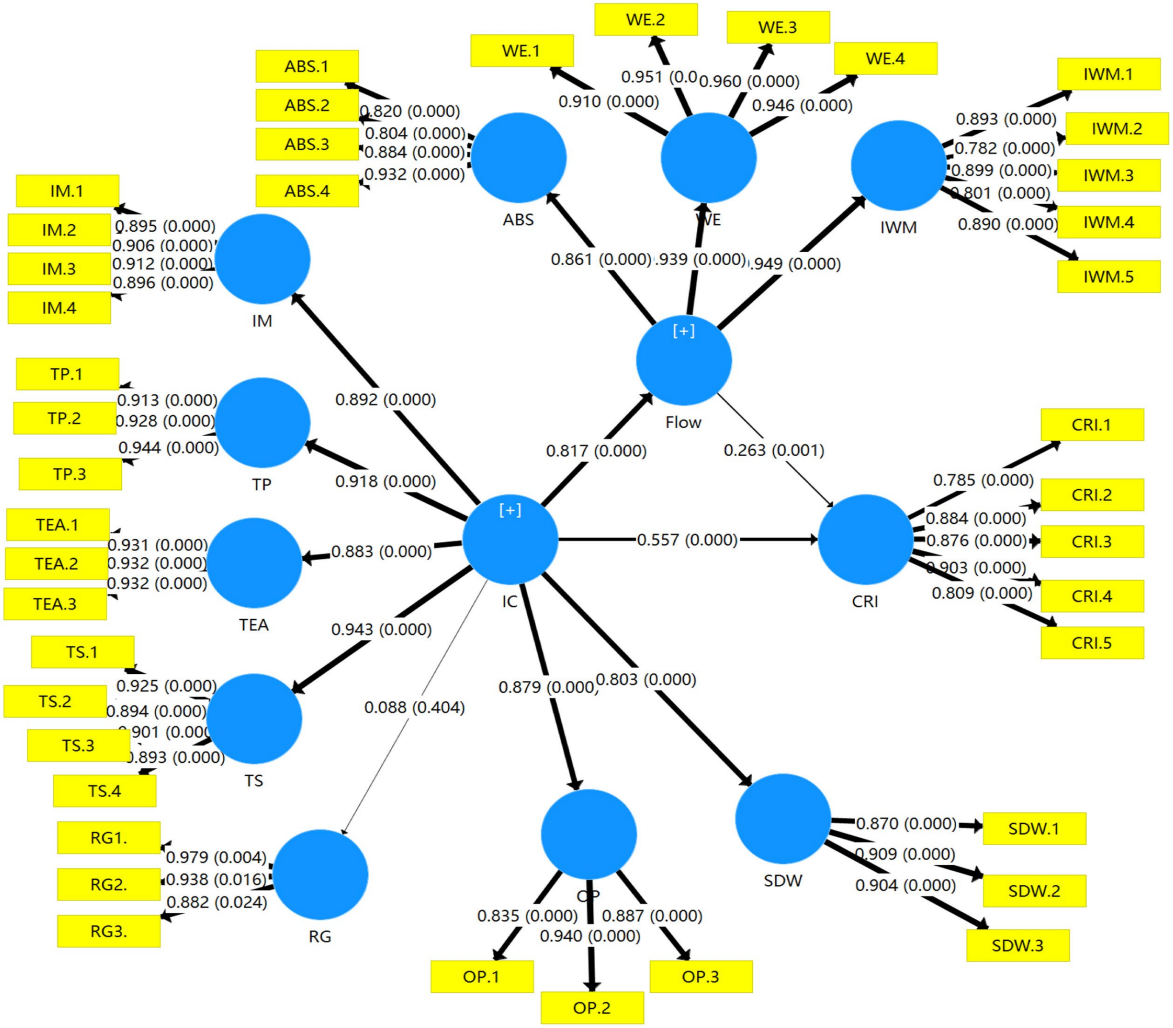
The results of PLS analysis.

We controlled for gender, class, and weekly class hour variables to avoid their potential effects on creative role identity. A PLS bootstrapping algorithm was used to obtain the saliency results. Smart-PLS was used to analyze data, and the basic information of subsamples was set at a larger number of 5,000 to obtain conventional PLS estimates with bootstrap significance. Figure 1 shows the results of the model. The study shows that in the proposed model, IC can explain 66.7% of the change in flow (*R*^2^ = 66.7%); while the explanatory power of IC and flow for the change in CRI is 61.8%(*R*^2^ = 61.8%).

According to the PLS analysis results, IC was positively related to flow (*β* = 0.834, *p* = 0.000), IC was significantly related to CRI (*β* = 0.571, *p* = 0.000), and flow significantly affected CRI (*β* = 0.268, *p* = 0.001). Hence, all the hypotheses were strongly supported. Table 2 shows these values.

Table 4 shows the total effects of flow and IC on CRI. Table 4 indicates that IC had a larger total effect on CRI than flow did, as IC had an indirect effect on CRI. The mediating effect of flow between IC and CRI was proven. This result provides important implications for colleges to conduct educational reforms.

**Table 4:** Direct, indirect, and total effects of each factor on creative role identity.

	Flow (direct effect)	CRI (direct effect)	CRI (indirect effect)	CRI (total effect)
Flow		0.263^**^		0.263^***^
IC	0.817^***^	0.557^***^	0.215	0.771^***^

** *p < 0.01, and*

*** *p < 0.001*.

## Discussion

### Discussion of Results

This study links the impact of innovation climate on creative role identity in universities with flow and brings interdisciplinary contributions to innovation education and innovation talent cultivation. A questionnaire survey was conducted on 226 students from 6 classes of a vocational university selected for this study. The empirical results support the two hypotheses of this paper well. The main conclusions of this study are as follows:

The innovation climate has a significant positive impact on the identity of individual creative roles.

1. The research results support the innovation climate created by colleges and universities through innovation and entrepreneurship activities and course design, which can stimulate students’ creative role identity. The innovation climate is an important antecedent that affects the creative role identity and then affects the willingness to innovate and the behavior of innovation.
2. The main contribution of flow is to mediate the positive effect of innovation climate on creative role identity. It verifies the view of situational strength theory that the environment influences intentions and behaviors through individual psychological factors. This model helps to explain how the innovation climate can better influence students’ individual creativity in university innovation education and provides new ideas for classroom organization.

### Theoretical Contributions

In general, from the perspective of situational strength theory, this study analyzes the influence mechanism and individual psychological mechanism of the influence of innovation climate on individual creative role identity. The theoretical contributions of this research are as follows:

First, we found that previous research suggested that creative role identity can drive individuals to produce innovative behaviors. However, few studies have investigated the antecedents and motivational mechanisms of creative role identity. Our research addresses this important gap by linking school innovation climates with a student’s creative role identity. We introduce situational strength theory to study the antecedents of creative role identity and regard innovation climate as a psychological impact, which provides a new perspective for the research of creative role identity and extends the understanding of the antecedents of creative role identity.

Second, this study proposes and verifies the influence mechanism of the external environment on creative role identity, discovers the key role of flow as a psychological mechanism in this process, and unpacks an important mechanism linking innovation climate to creative role identity. Previous studies have noticed a significant positive correlation between innovation climate and individual creative behavior, but the detailed psychological mechanism has not been explored in depth. Using role identity theory and situational strength theory, we adopt a new theoretical framework to explain the mediating role of flow between innovation climate and creative role identity and point out that fully engaged and highly excited of mental state is an important way to connect the external environment and role identity. It is helpful to open the “black box” in the process of the influence of innovation climate on creative identity and enrich the theory of creativity research. Our research has found that if an organization wants an innovation climate to have a stronger positive effect on creative role identity and even innovative behavior, an effective way is to build a flow experience to stimulate individuals’ desire to learn and explore. This allows individuals to not only express their creativity but also to become more creative systematically and ultimately to attain creative achievement.

Third, this study is an extension of the research on the relationship between external climate and individual role identity in the context of pedagogy, providing useful new insights for educational methods. In the teaching activities of the project method, the innovation climate created by the school influences the students’ creative role identity through the flow classroom and then promotes the students to produce innovative behaviors and innovation results.

### Managerial Implications

Our research has important practical implications for university innovation education. In particular, the tide of innovation sweeping the world will eventually lead the development of creativity education to converge, that is, to cultivate talent with creative spirit and creative ability. Therefore, it has become an urgent task for universities to carry out in-depth innovation education reform and cultivate high-level innovative talent.

Our research has implications for the development of innovation educational methods in colleges and universities and the cultivation of innovative talent.

Implications for school administrators:

1. Schools should further create a supportive environment for innovation and entrepreneurship education, establish a variety of incentive mechanisms to encourage innovation, and provide abundant learning resources and incubation resources. For example, they could create a free creative innovation laboratory, a prototype production room, an exhibition area for creative works, etc., and build a business incubation base based on regional industries.
2. Integrate creativity-inducing training into the curricula, build a diverse innovation practice platform, and organize various forms of innovation and entrepreneurship community activities. For example, they could hold creative competitions with rewards at the end of a course to select the outcome and they could create an innovation climate through the extension of competition and feedback mechanisms inside and outside the classroom.
3. Better nurture teaching staff and cultivate innovative teachers while supporting teachers in innovating teaching methods, deepening the reform of classroom teaching, and creating a classroom climate with high student participation and vitality. For example, schools could establish an innovation education teacher club, develop a multistage teacher training system, and promote the formation of a dynamic mechanism among teachers to allow them to spontaneously participate in innovative teaching reform and research.

Creativity education in colleges and universities is an educational process in which teachers use creativity courses and creative education strategies to cultivate college students’ creative thinking ability and stimulate college students’ innovative behaviors. Implications for teachers:

1. Set clear goals and assign challenging tasks to enhance students’ intrinsic motivation for learning when developing courses, carrying out teaching design, and organizing teaching activities.
2. Ensure timely feedback of results, strengthen students’ active learning behavior, and improve students’ concentration and learning enthusiasm.
3. Improve analysis of the learning situation, balance the students’ skills and the difficulty of the challenge, and meet the personalized learning needs of students at different levels with learning tasks of different difficulty.
4. Adhere to the student-centered model, establish an open learning climate, encourage peers to learn from each other, take into account the differences between students, allow students to gain positive emotional experience, enjoy the learning process and enjoy the student-centered model, and support students as active constructive learners.
5. Build environment resources for active teaching that include features, such as virtual simulation in order to relieve students’ learning fatigue and enhance their learning motivation and interest.
6. Teachers should continuously improve their own innovative thinking and innovative ability, change the traditional paradigm of their teaching philosophy, and use new theories, new concepts, and new methods to improve themselves, to broaden and deepen their knowledge, display creative thinking, be able to use creative techniques to carry out creative teaching reform. Ultimately, developing these skills can help teachers fully demonstrate a winning personality in innovation education, gain the love of students, build a harmonious relationship between teachers and students, and play an exemplary role.

### Research Limitations and Future Research

The limitations of this study and future research directions are as follows: (1) Selected sample of this study was the researchers’ students. Although the proportion of engineering and liberal arts majors was considered, the limitations of convenient sampling still exist. When conditions are available, stratified sampling can be adopted in universities and vocational colleges nationwide to conduct more complete research. (2) More diverse research samples are needed. The research sample consisted of university students. Although the sample group was suitable for the main research purpose, there are still some differences between the characteristics of university students and employees at enterprises. Future research should continue to take other samples (such as employees at enterprises) to verify the validity of the model and increase the external validity of the research conclusions. (3) When analyzing the effect of innovation climate on creative role identity, this study focused on the mediating role of flow. To further explore how the effect of innovation climate on creative role identity changes under different mediating variables and open the “black box” of the relationship between the two, future research can introduce other classical mediator variables for analysis and build multiple models.

## Data Availability Statement

The original contributions presented in the study are included in the article/supplementary material, further inquiries can be directed to the corresponding author.

## Ethics Statement

Written informed consent was obtained from the individual(s) for the publication of any potentially identifiable images or data included in this article.

## Author Contributions

BD and JW designed the study, performed the experiments, and wrote the manuscript. JH and JC performed the experiments and analyzed the data. All authors contributed to the article and approved the submitted version.

## Funding

This work was supported by Innovation and Entrepreneurship Project of Guangzhou Education Bureau (2020KC018 and 2019PT102), Youth Innovation Talents Project of Guangdong Provincial Department of Education (2019GWQNCX081), and Characteristic Innovation Projects of Colleges and Universities in Guangdong Province (2018GWTSCX057).

## Conflict of Interest

The authors declare that the research was conducted in the absence of any commercial or financial relationships that could be construed as a potential conflict of interest.

## Publisher’s Note

All claims expressed in this article are solely those of the authors and do not necessarily represent those of their affiliated organizations, or those of the publisher, the editors and the reviewers. Any product that may be evaluated in this article, or claim that may be made by its manufacturer, is not guaranteed or endorsed by the publisher.

## Abbreviations

CRI: Creative Role Identity
IC: Innovation Climate
TS: Teacher Support
IWM: Intrinsic Work Motivation
ABS: Absorption
TEA: Teamwork
WE: Work Enjoyment
IM: Incentive Mechanism
OP: Organization Promotion
SDW: Self-Directed Work
RG: Resource Guarantee
TP: Teacher Practice
